# Latent class analysis of intrinsic capacity and its association with quality of life in hospitalized older people with stroke: a cross-sectional study

**DOI:** 10.3389/fpubh.2026.1905197

**Published:** 2026-10-12

**Authors:** Xin-Xin Tang, Qian-jie Wu, Xiao-Min Zhang, Qiu-Yue Tang, Ling Yu

**Affiliations:** 1 Department of Neurology, The First Affiliated Hospital with Nanjing Medical University, Nanjing, Jiangsu, China; 2 Department of Nursing, The Affiliated Suqian First People's Hospital of Nanjing Medical University, Suqian, Jiangsu, China

**Keywords:** intrinsic capacity, latent class analysis, neurology, quality of life, stroke

## Abstract

**Objective:**

This study aimed to identify latent classes of intrinsic capacity among hospitalized older people with stroke and to examine the associations of these classes with stroke-specific quality of life.

**Methods:**

A cross-sectional design was used, and 314 hospitalized older people with stroke were consecutively recruited. Stroke severity was assessed using the National Institutes of Health Stroke Scale (NIHSS), comorbidity burden using the Age-adjusted Charlson Comorbidity Index (ACCI), perceived social support using the Perceived Social Support Scale (PSSS), intrinsic capacity across five domains using the Intrinsic Capacity Assessment Tool, and stroke-specific quality of life using the Stroke-Specific Quality of Life Scale (SS-QOL). Latent class analysis identified intrinsic-capacity profiles, and multivariable logistic regression was used to examine factors associated with latent-class membership, and ANOVA was used to compare SS-QOL scores across the identified classes.

**Results:**

LCA identified three latent classes of intrinsic capacity among hospitalized older people with stroke: the low motor–low vitality group (27.70%), the low cognitive–moderate vitality, psychological, and sensory group (17.20%), and the function maintenance group (55.10%). Multinomial logistic regression showed that age, stroke severity, sleep duration, and social support were associated with membership in the low motor–low vitality group, whereas education level was associated with membership in the low cognitive–moderate vitality, psychological, and sensory group. Significant differences were observed in most stroke-specific quality-of-life domains across the identified groups (*p* < 0.05).

**Conclusion:**

Three distinct intrinsic-capacity profiles were identified among hospitalized older people with stroke, and these profiles were associated with different levels of stroke-specific quality of life. Both clinical and psychosocial factors were associated with profile membership, highlighting the need for targeted interventions to preserve intrinsic capacity and improve quality of life.

## 1 Introduction

Stroke remains the second leading cause of death and long-term disability worldwide and in China and has been identified as a priority chronic disease for prevention and control (1, 2). The burden of stroke increases substantially with age, making older adults a particularly vulnerable population (3). Beyond mortality and disability, stroke has a profound and lasting impact on quality of life (QOL), including physical function, mood, cognition, and social participation, even beyond the acute stage (4, 5).

In 2015, the World Health Organization (WHO) introduced the concept of intrinsic capacity (IC) in the World Report on Aging and Health (6). IC refers to the composite of all physical and mental capacities that an individual can draw on at a given time and includes five core domains: locomotion, vitality, sensory function, cognition, and psychological wellbeing (7). Decline in IC has been associated with poor quality of life, disability, frailty, dependence, and other adverse health outcomes in older adults (8–10). However, much of the existing literature has focused on single functional domains rather than multidimensional IC patterns (6, 10).

Recently, person-centered analytical approaches have been increasingly used to identify multidomain IC profiles in older populations. For example, latent class analysis (LCA) has been used to classify heterogeneous patterns of IC impairment and to explore factors associated with class membership (11, 12). Nevertheless, evidence remains limited regarding IC profiles specifically among hospitalized older people with stroke. This issue is clinically important because people with stroke often present with marked heterogeneity in motor, cognitive, sensory, nutritional, and psychological status, as well as in stroke severity, comorbidity burden, sleep, and social support (13, 14). In addition, major stroke rehabilitation guidelines emphasize multidisciplinary, patient-centered care and individualized goal setting across physical, cognitive, emotional, and participation domains (15).

LCA is a probability-based statistical approach that can identify unobserved subgroups with similar response patterns and therefore provides a useful framework for characterizing multidimensional IC heterogeneity (16). To our knowledge, few studies have applied LCA to examine IC profiles specifically in hospitalized older people with stroke and further linked these profiles to stroke-specific quality of life. Therefore, the aims of this study were to identify latent classes of IC in hospitalized older people with stroke, examine factors associated with class membership, and explore differences in stroke-specific quality of life across these classes. The findings may provide evidence for more individualized rehabilitation and nursing strategies based on domain-specific risk patterns.

## 2 Methods

This cross-sectional study was reported in accordance with the Strengthening the Reporting of Observational Studies in Epidemiology (STROBE) statement (17).

### 2.1 Study participants

Participants were consecutively recruited from May 2024 to November 2024 from the Department of Neurology, Jiangsu Province Hospital (the First Affiliated Hospital of Nanjing Medical University). Eligible participants met all of the following inclusion criteria: (1) diagnosis of ischemic or hemorrhagic stroke according to the Key Points for Diagnosis of Major Cerebrovascular Diseases in China (2019), confirmed by brain computed tomography (CT) or magnetic resonance imaging (MRI) (18); (2) age ≥60 years; and (3) clinically stable stroke beyond the acute stage, as judged by the treating neurologist on the basis of the medical record and current clinical condition, including patients in the recovery or sequelae stage. To ensure clinical stability, enrollment was performed at least 48 h after admission, when the patient had stable vital signs and no acute deterioration.

Exclusion criteria were as follows: (1) severe cognitive impairment or a history of mental illness; and (2) comorbid cancer or vital organ failure. Severe cognitive impairment was screened at enrollment by trained investigators using the Chinese version of the Mini-Mental State Examination (MMSE), together with an assessment of communication ability and response capacity during the enrollment interview. Participants were excluded if they were unable to understand the study information or interview questions, unable to communicate meaningfully with the investigator, or unable to provide coherent and consistent responses sufficient to complete the questionnaire assessment.

Sample size estimation followed the variable multiple method, yielding a theoretical sample size range of 160–320 (16 variables × 10–20) (18). After allowing for a potential 10% rate of questionnaire loss or invalidity, the minimum required sample size was set at 178.

### 2.2 Investigation tools

The main study outcomes were intrinsic capacity (five-domain status and total score) and stroke-specific quality of life. Additional measured variables included neurological deficit severity, comorbidity burden, and perceived social support. The instruments were selected to align with the WHO intrinsic capacity framework and the study objectives, and ensure feasibility for bedside assessment in hospitalized older people with stroke. All measures are widely used in stroke/geriatric research and were feasible for bedside assessment in hospitalized older people with stroke.

#### 2.2.1 General information questionnaire

A self-designed questionnaire was administered to collect demographic data (gender, age, body mass index [BMI], education level, marital status), socioeconomic status (employment situation, medical insurance type, residence), lifestyle factors (smoking, alcohol consumption, sleep duration), and stroke-related characteristics (stroke type, stroke severity, limb muscle strength). Limb muscle strength was assessed using the Lovett grading system (0–5). “Normal” was defined as grade 5 in all limbs; “abnormal” was defined as grade ≤ 4 in any limb on either side. Spasticity was not assessed (e.g., Modified Ashworth Scale was not collected).

#### 2.2.2 National Institutes of Health Stroke Scale

Developed in 1989, the National Institutes of Health Stroke Scale (NIHSS) assesses neurological function using 11 items, including level of consciousness, visual fields, limb motor function, sensation, and language (19). Scores range from 0 to 42, with 0 to 1 defined as normal, 2–4 as mild, 5–15 as moderate, and >15 as severe. The NIHSS is a well-validated instrument for quantifying neurological deficit severity in people with stroke (20). The Cronbach's α coefficient for this scale is 0.810.

#### 2.2.3 Age-adjusted Charlson Comorbidity Index

The Age-adjusted Charlson Comorbidity Index (ACCI) is a structured scoring instrument designed to quantify the complexity of patients' clinical conditions and to prospectively estimate mortality risk in those with chronic diseases and cancer (21). This index integrates 19 clinically relevant diseases, each assigned a specific weight according to its potential impact on mortality. The scoring system is based on two primary dimensions. The first dimension, age-stratified scoring, assigns points as follows: 40–49 years, 1 point; 50–59 years, 2 points; 60–69 years, 3 points; and 70 years or older, 4 points. The second dimension, disease burden, accounts for the weighted scores of the 19 specified comorbid conditions. Data on pre-existing comorbidities prior to stroke diagnosis were obtained from medical record review. The cumulative score reflects the overall comorbidity burden, with scores ≥5 indicating multiple comorbidities (high burden) and scores < 5 indicating non-multiple comorbidities. The CCI/ACCI has been extensively evaluated, and evidence supporting its clinimetric properties has been summarized in a critical review (21). The Cronbach's α coefficient for the ACCI is 0.832.

#### 2.2.4 Perceived Social Support Scale

The Perceived Social Support Scale (PSSS) measures multidimensional self-perceived social support (22). It consists of 12 items across three domains: family support (items 3, 4, 8, 11), friend support (items 6, 7, 9, 12), and other support (items 1, 2, 5, 10). Items are rated on a 7-point scale (1–7), with total scores ranging from 12 to 84. Scores of 12–36 indicate low social support, 37–60 indicate moderate social support, and 61–84 indicate high social support. The PSSS has been validated and has shown acceptable psychometric properties in Chinese hospitalized populations (22). The Cronbach's α coefficient for this scale is 0.840.

#### 2.2.5 Intrinsic capacity assessment tools

Intrinsic capacity was assessed according to the World Health Organization's five-domain framework (7). For latent class analysis, each domain was dichotomized as preserved (1) or impaired (0) based on predefined criteria.

Vitality: assessed using the Mini-Nutritional Assessment Short-Form (MNA-SF) (23). Scores < 11 were classified as impaired vitality (risk of malnutrition or malnutrition), whereas scores ≥11 were classified as preserved vitality. The MNA-SF is a widely used and validated screening tool for nutritional status in older people (23). The Cronbach's α coefficient in this study was 0.816.

Psychological: assessed using the 15-item Geriatric Depression Scale (GDS-15) (24). Scores ≥8 indicated impaired psychological status, whereas scores < 8 indicated preserved psychological status. The Chinese version of the GDS-15 has demonstrated acceptable psychometric properties in older Chinese populations (24). The Cronbach's α coefficient in this study was 0.793.

Cognition: assessed using the Mini-Mental State Examination (MMSE). Education-specific cutoffs were used to define cognitive impairment: ≤ 17 for illiterate participants, ≤ 20 for those with primary school education, and ≤ 24 for those with junior high school education or above (25). Scores above these thresholds were classified as preserved cognition. The Chinese version of the MMSE was used, and measurement invariance has been examined in older Chinese populations (25). The Cronbach's α coefficient in this study was 0.838.

Locomotion (motor): assessed using the Short Physical Performance Battery (SPPB), which includes standing balance, 4-m walking speed, and the five-times sit-to-stand test (26, 27). Scores ≤ 8 indicated impaired locomotion, whereas scores >8 indicated preserved locomotion. The SPPB is a validated measure of lower-extremity physical performance and has been widely used in older-adult populations (27). The Cronbach's α coefficient in this study was 0.812.

Sensory: assessed using visual acuity and whisper tests (28). Sensory function was classified as impaired if the participant had abnormal visual acuity and/or an abnormal whisper test result; otherwise, it was classified as preserved. Normal visual function was defined as uncorrected bilateral visual acuity ≥0.8.

The total intrinsic capacity score ranged from 0 to 5, with higher scores indicating better overall intrinsic capacity.

#### 2.2.6 Stroke-specific quality of life scale

The Stroke-Specific Quality of Life Scale (SS-QOL) was employed to assess quality of life in people with stroke (29). It comprises 49 items across 12 domains: vision, language, mood, cognition, upper extremity function, personality, mobility, family roles, self-care, energy, work productivity, and social roles. Each item is rated on a 5-point scale, with higher total scores indicating better quality of life. This instrument has demonstrated acceptable validity and reliability in previous studies (30, 31). The Cronbach's α coefficient is 0.832.

### 2.3 Data collection and quality control

This study was approved by the Ethics Review Committee of the First Affiliated Hospital of Nanjing Medical University (approval number: 2024-SR-100). All participants provided written informed consent before the interview-based assessment. Data collection was conducted by two graduate nursing students who had received standardized training before the study, including training in questionnaire administration, communication procedures, and assessment consistency.

After informed consent was obtained, trained investigators conducted structured, interviewer-administered, one-to-one assessments using standardized paper questionnaires. Before formal data collection, participants' ability to understand the interview content and provide reliable responses was verified during enrollment screening. Demographic and clinical information was primarily obtained from the hospital electronic medical record system, and missing items were supplemented through direct inquiry of participants or family members whenever possible.

Eligibility screening was completed before questionnaire administration. A total of 322 eligible participants were enrolled and underwent the interviewer-administered assessment. Eight questionnaires were subsequently excluded in their entirety because of invalid or incomplete responses, resulting in 314 valid questionnaires and an effective response rate of 97.5%.

### 2.4 Statistical analysis

Data entry followed a double-independent process with cross-verification. Latent class models were constructed using Mplus version 8.0 (Muthén & Muthén, Los Angeles, CA, United States) Model fit was evaluated using the Akaike Information Criterion (AIC), Bayesian Information Criterion (BIC), and sample-size adjusted BIC (aBIC), with lower values indicating better fit. The optimal number of classes was determined using the Lo-Mendell-Rubin adjusted likelihood ratio test (LMR-LRT) and bootstrap likelihood ratio test (BLRT), with *p* < 0.05 indicating that the k-class model fit significantly better than the (k−1)-class model. Classification accuracy was assessed using entropy, with values >0.80 indicating good classification quality (32).

Statistical analyses were performed using SPSS version 27.0 (IBM Corp., Armonk, NY, United States). Normality of continuous variables was assessed using the Shapiro–Wilk test together with visual inspection of histograms and Q–Q plots. Homogeneity of variances was examined using Levene's test before ANOVA. Normally distributed data are presented as mean ± standard deviation and were compared using one-way analysis of variance (ANOVA), followed by least significant difference (LSD) *post-hoc* tests where appropriate. Skewed data are presented as median (interquartile range) and were compared using non-parametric tests. Categorical variables are described as frequencies and percentages and were compared using the chi-square test.

To examine factors associated with latent-class membership, multinomial logistic regression was performed with the Function Maintenance Group as the reference category. Candidate predictors were selected based on both their univariate association with latent-class membership and their theoretical or clinical relevance to intrinsic capacity in older people with stroke, as informed by previous literature and the study objectives. Variables entered into the model included age group, education level, sleep duration, stroke severity, and perceived social support. For regression analysis, education level was recategorized into three groups (primary school or below, junior high school, and senior high school or above), and sleep duration was dichotomized as >6 h vs. ≤ 6 h. Categorical predictors were dummy coded, and reference categories were specified in Table 1. Multicollinearity among independent variables was assessed using variance inflation factors (VIFs), and no serious multicollinearity was detected. Odds ratios (ORs), 95% confidence intervals (CIs), and *p*-values are reported for all model estimates. A two-sided *p*-value < 0.05 was considered statistically significant.

**Table 1:** Multivariate logistic regression analysis of factors associated with latent classes of intrinsic capacity.

Predictor	Reference category	Low motor–low vitality group vs. function maintenance group OR (95% CI)	*p*	Low cognitive–moderate vitality, psychological and sensory group vs. function maintenance group OR (95% CI)	*p*
Age 60–74 years	≥75 years	0.365 (0.185–0.718)	0.004	0.452 (0.204–1.002)	0.051
Mild stroke severity	Moderate/severe stroke	0.158 (0.064–0.393)	< 0.001	0.965 (0.233–3.987)	0.960
Low social support	High social support	3.420 (1.158–10.103)	0.026	0.560 (0.112–2.808)	0.481
Moderate social support	High social support	1.994 (1.070–3.717)	0.030	0.639 (0.303–1.346)	0.239
Primary school or below	Senior high school or above	0.643 (0.285–1.453)	0.289	0.191 (0.043–0.859)	0.031
Junior high school	Senior high school or above	1.595 (0.833–3.054)	0.159	1.712 (0.870–3.370)	0.120
Sleep duration >6 h	≤ 6 h	0.424 (0.231–0.779)	0.006	0.526 (0.271–1.021)	0.058

Multinomial logistic regression was performed with latent class membership as the dependent variable. The Function Maintenance Group was used as the reference outcome category. Reference categories for predictors were as follows: age ≥75 years, moderate/severe stroke, high social support, senior high school or above, and sleep duration ≤ 6 h. OR, odds ratio; CI, confidence interval.

Homogeneity of variances across latent classes was examined using Levene's test before one-way ANOVA. For SS-QOL domains with homogeneous variances, one-way ANOVA followed by least significant difference (LSD) *post-hoc* comparisons was used. For domains with unequal variances, pairwise comparisons were interpreted using the Games–Howell procedure. Pairwise comparison results are reported as mean differences, 95% confidence intervals (CIs), and *p*-values. No additional adjustment for multiple testing across SS-QOL domains was applied.

After the optimal latent class model was selected in Mplus, each participant was assigned to the class with the highest posterior probability (most likely class membership). This assigned class variable was then exported to SPSS and used in subsequent analyses, including chi-square tests, one-way ANOVA, and multinomial logistic regression. Classification uncertainty was not directly incorporated into these downstream analyses.

## 3 Results

### 3.1 General information

A total of 314 older people with stroke were included in this study, consisting of 207 males and 107 females. Participant age ranged from 60 to 86 years, with a mean ± standard deviation of 68.97 ± 6.36 years. Additional demographic and clinical information are presented in Table 2.

**Table 2:** Demographic and clinical characteristics of older adults with stroke.

Variable	Category	Number	Percentage (%)
Gender	Male	207	65.9
	Female	107	34.1
Age	60–74years	249	79.3
	≥75years	65	20.7
BMI	< 18.5	11	3.5
	18.5–24.9	183	58.3
	≥25	120	38.2
Educational level	Illiterate	18	5.7
	Primary school	36	11.5
	Junior high school	93	29.6
	Senior high school/technical secondary school	95	30.2
	College or above	72	22.9
Marital status	Married	296	94.2
	Single	9	2.9
	Divorced/widowed	9	2.9
Employment situation	Employed	53	16.9
	Unemployed/retired	261	83.1
Medical insurance type	Employee medical insurance	120	38.2
	Resident medical insurance	10	3.2
	Non-local medical insurance	168	53.5
	Self-pay	16	5.1
Residence	Urban	219	69.7
	Rural	95	30.3
Smoking	Yes	80	25.5
	Never/quit	234	74.5
Alcohol consumption	Yes	82	26.1
	Never/quit	232	73.9
Sleep duration	>6 h	160	51.0
	4–6 h	60	19.1
	< 4 h	94	29.9
Stroke type	Hemorrhagic stroke	60	19.1
	Ischemic stroke	254	80.9
Limb muscle strength (Lovett grade)^a^	Normal	235	74.8
	Abnormal	79	25.2
Stroke severity (NIHSS)	Mild stroke	281	89.5
	Moderate/severe stroke	33	10.5
ACCI	Multiple comorbidities	109	34.7
	Non-multiple comorbidities	205	65.3
PSSS	Low social support	22	7.0
	Moderate social support	118	37.6
	High social support	174	55.4

^a^Limb muscle strength was assessed using the Lovett grading system (0–5). Normal = grade 5 in all limbs; abnormal = grade ≤ 4 in any limb on either side.

BMI, body mass index; NIHSS, National Institutes of Health Stroke Scale; ACCI, Age-adjusted Charlson Comorbidity Index; PSSS, Perceived Social Support Scale.

### 3.2 LCA of intrinsic capacity in older people with stroke

The median intrinsic capacity score of older people with stroke was 4 (interquartile range: 3–5). A decline in at least one dimension of intrinsic capacity was observed in 72.0% (226 cases) of people with stroke. Among the five dimensions, decline in the vitality dimension was most common (44.9%, 141 cases), followed by motor (32.5%, 102 cases), sensory (29.6%, 93 cases), cognitive (29.0%, 91 cases), and psychological (12.4%, 39 cases) dimensions. LCA was performed using the five dimensions as manifest variables. Although AIC, BIC, and sample-size aBIC values decreased as the number of classes increased, and the BIC reached its lowest value in the two-class model, the three-class model achieved the optimal entropy value, indicating superior classification accuracy. Furthermore, the Lo-Mendell-Rubin (LMR) and Bootstrap Likelihood Ratio Test (BLRT) results were statistically significant (*p* < 0.05), confirming that the three-class model was significantly better than the two-class model (Table 3). Comprehensive evaluation of multiple fit indices confirmed the three-class model as optimal. The average posterior probabilities for the three classes were 0.879, 0.976, and 1.000, respectively, demonstrating high stability and reliability of classification. Based on the highest posterior probability, participants were assigned to one of the three latent classes for subsequent analyses. Figure 1 presents the conditional probability distribution from the Mplus software analysis, illustrating the characteristics of the three latent classes of intrinsic capacity. Class 1 (C1): Low Motor–Low Vitality Group–87 participants (27.7%) with markedly reduced motor and vitality dimensions; Class 2 (C2): Low Cognitive–Moderate Vitality, Psychological, and Sensory Group–54 participants (17.2%) with significant decline in cognitive function and moderate impairment in vitality, psychological, and sensory dimensions; Class 3 (C3): Function Maintenance Group–173 participants (55.1%) with relatively preserved functional dimensions.

**Table 3:** Fit indices for latent class analysis models of intrinsic capacity.

Model	K	AIC	BIC	aBIC	LMR (P)	BLRT (P)	Entropy	Class probability (%)
Model 1	5	1833.209	1851.956	1836.098	–	–	–	100
Model 2	11	1774.943	1816.187	1781.298	< 0.001	< 0.001	0.514	51.59/48.41
Model 3	17	1770.463	1834.203	1780.284	< 0.001	< 0.001	0.907	27.70/17.20/55.10
Model 4	23	1770.124	1856.36	1783.411	0.2093	0.1304	0.894	1.91/21.98/17.20/58.91
Model 5	29	1774.919	1883.651	1791.671	0.5995	0.2667	0.796	14.33/19.74/6.69/2.23/57.01

**Figure 1:**
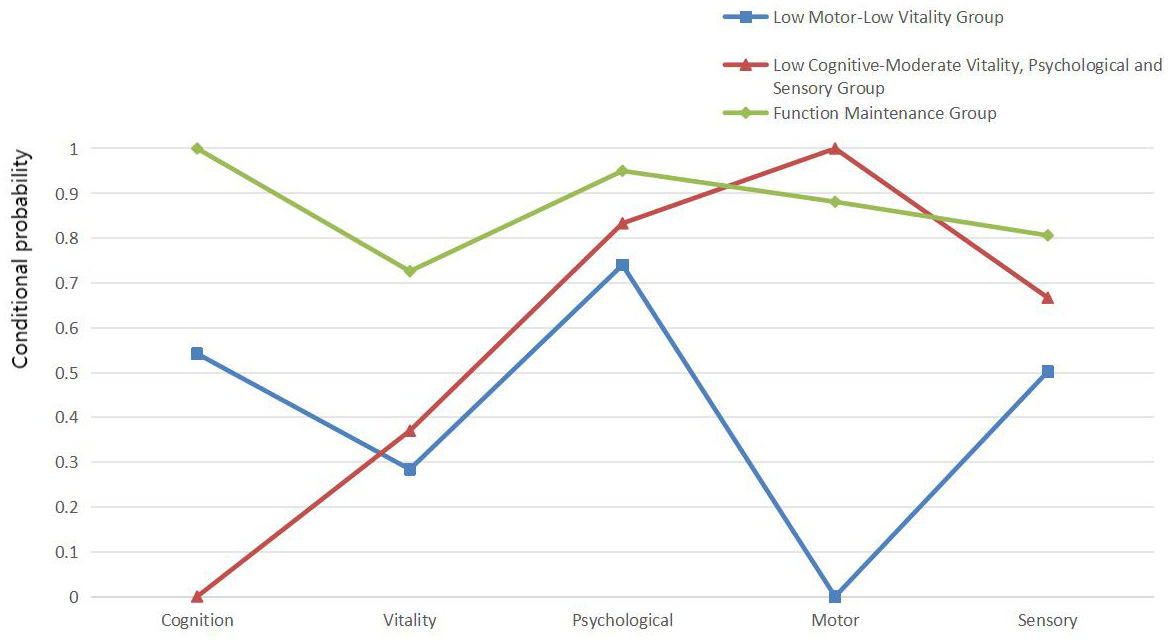
Conditional probability profiles of three latent classes of intrinsic capacity.

### 3.3 Comparison of characteristics among older people with stroke in different latent classes

Comparison of demographic and clinical characteristics among the three latent classes demonstrated no statistically significant differences in gender, BMI, marital status, employment status, residence, smoking, alcohol consumption, or stroke type (all *p* > 0.05). Variables with significant differences are presented in Table 4.

**Table 4:** Comparison of demographic and clinical variables across latent classes of intrinsic capacity.

Variable	Category	Low motor-low vitality group (*n* = 87)	Low cognitive-moderate vitality, psychological and sensory group (*n* = 54)	Function maintenance group (*n* = 173)	Statistic	*P*
Age [*n* (%)]	60–74 years	58 (66.7)	41 (75.9)	150 (86.7)	X2 = 14.612	< 0.001
	≥75 years	29 (33.3)	13 (24.1)	23 (13.3)		
Educational level [*n* (%)]	Illiterate	6 (6.9)	0 (0.0)	12 (6.9)	X2 = 17.862	0.022
	Primary school	7 (8.0)	2 (3.7)	27 (15.6)		
	Junior high school	28 (32.2)	22 (40.7)	43 (24.9)		
	High school/Technical secondary school	28 (32.2)	21 (38.9)	46 (26.6)		
	College and above	18 (20.7)	9 (16.7)	45 (26.0)		
Medical insurance type [*n* (%)]	Employee medical insurance	26 (29.9)	23 (42.6)	71 (41.0)	X2 = 14.351	0.026
	Resident medical insurance	4 (4.6)	0 (0.0)	6 (3.5)		
	Non-local medical insurance	47 (54.0)	30 (55.6)	91 (52.6)		
	Self-pay	10 (11.5)	1 (1.9)	5 (2.9)		
Limb muscle strength (Lovett grade)^a^ [*n* (%)]	Normal	47 (54.0)	42 (77.8)	146 (84.4)	X2 = 28.665	< 0.001
	Abnormal	40 (46.0)	12 (22.2)	27 (15.6)		
Sleep duration [*n* (%)]	>6 h	33 (37.9)	22 (40.7)	105 (60.7)	X2 = 19.397	< 0.001
	4–6 h	17 (19.5)	10 (18.5)	33 (19.1)		
	< 4 h	37 (42.5)	22 (40.7)	35 (20.2)		
Stroke severity (NIHSS) [*n* (%)]	Mild	65 (74.7)	51 (94.4)	165 (95.4)	X2 = 27.982	< 0.001
	Moderate/severe	22 (25.3)	3 (5.6)	8 (4.6)		
ACCI [*n* (%)]	Multiple comorbidities	40 (46.0)	19 (35.2)	50 (28.9)	X2 = 7.454	0.024
	Non-multiple comorbidities	47 (54.0)	35 (64.8)	123 (71.1)		
PSSS [*n* (%)]	Low support level	8 (9.2)	2 (3.7)	12 (6.9)	X2 = 12.176	0.016
	Medium support level	42 (48.3)	13 (24.1)	63 (36.4)		
	High support level	37 (42.5)	39 (72.2)	98 (56.6)		

^a^Normal = Lovett grade 5 in all limbs; abnormal = grade ≤ 4 in any limb on either side.

NIHSS, National Institutes of Health Stroke Scale (higher scores indicate more severe stroke); ACCI, Age-adjusted Charlson Comorbidity Index (higher scores indicate greater comorbidity burden); PSSS, Perceived Social Support Scale (higher scores indicate higher perceived social support).

Data are presented as *n* (%). Group comparisons were conducted using the chi-square test. Significant *p*-values are shown in bold (*P* < 0.05).

### 3.4 Association between latent classes of intrinsic capacity and quality of life scores

One-way ANOVA was conducted to compare SS-QOL scores across the three latent classes. Significant between-class differences were observed for all domains except personality (*F* = 1.487, *p* = 0.228). Levene's test indicated unequal variances for vision, language, mood, mobility, self-care, upper extremity function, and work productivity; therefore, Games–Howell *post-hoc* tests were used for these domains. LSD *post-hoc* tests were used for the remaining domains with homogeneous variances. Compared with the Function Maintenance Group, the Low Motor–Low Vitality Group had significantly lower scores in vision, language, mood, cognition, upper extremity function, mobility, family roles, self-care, energy, work productivity, and social roles, whereas personality did not differ significantly. Compared with the Function Maintenance Group, the Low Cognitive–Moderate Vitality, Psychological, and Sensory Group had significantly lower scores in cognition, family roles, and social roles, whereas the remaining domains did not differ significantly. Detailed results, including Levene's test results, mean differences, 95% confidence intervals, and *p*-values for pairwise comparisons, are presented in Table 5.

**Table 5:** A quality of life scores across latent classes of intrinsic capacity.

Dimension	Score range	Low motor–low vitality group (*n* = 87)	Low cognitive–moderate vitality, psychological, and sensory group (*n* = 54)	Function maintenance group (*n* = 173)	Levene's p	*F*	*P*	*Post-hoc* method	Low motor vs. function maintenance MD (95% CI), *p*	Low cognitive vs. function maintenance md (95% CI), *p*	Low motor vs. low cognitive MD (95% CI), *p*
Vision	3–15	13.00 ± 2.040	14.06 ± 1.089	14.35 ± 1.009	< 0.001	27.692	< 0.001	Games–Howell	−1.347 (−1.90, −0.80), < 0.001	−0.291 (−0.69, 0.11), 0.195	−1.056 (−1.68, −0.43), < 0.001
Language	5–25	22.30 ± 3.438	23.61 ± 2.791	24.21 ± 1.575	< 0.001	17.690	< 0.001	Games–Howell	−1.909 (−2.83, −0.99), < 0.001	−0.597 (−1.55, 0.36), 0.298	−1.312 (−2.57, −0.06), 0.038
Mood	5–25	20.56 ± 4.037	21.28 ± 5.163	22.34 ± 3.319	0.005	6.366	0.002	Games–Howell	−1.778 (−2.96, −0.59), 0.002	−1.063 (−2.85, 0.73), 0.334	−0.715 (−2.68, 1.25), 0.663
Cognition	3–15	9.44 ± 3.190	9.15 ± 3.024	11.65 ± 3.128	0.741	21.634	< 0.001	LSD	−2.216 (−3.03, −1.41), < 0.001	−2.505 (−3.46, −1.55), < 0.001	0.289 (−0.78, 1.35), 0.595
Upper extremity function	5–25	20.82 ± 4.285	23.83 ± 2.759	24.39 ± 1.437	< 0.001	50.473	< 0.001	Games–Howell	−3.577 (−4.70, −2.45), < 0.001	−0.560 (−1.50, 0.38), 0.331	−3.017 (−4.42, −1.61), < 0.001
Personality	3–15	11.37 ± 3.275	12.06 ± 3.253	12.06 ± 3.091	0.649	1.487	0.228	LSD	−0.690 (−1.51, 0.13), 0.099	0.002 (−0.97, 0.97), 0.996	−0.688 (−1.77, 0.39), 0.211
Mobility	6–30	21.86 ± 4.666	28.72 ± 1.676	28.47 ± 2.911	< 0.001	125.084	< 0.001	Games–Howell	−6.606 (−7.90, −5.31), < 0.001	0.254 (−0.50, 1.01), 0.704	−6.860 (−8.17, −5.56), < 0.001
Family roles	3–15	10.15 ± 2.751	10.80 ± 3.129	12.11 ± 2.723	0.207	15.381	< 0.001	LSD	−1.960 (−2.69, −1.24), < 0.001	−1.314 (−2.17, −0.45), 0.003	−0.647 (−1.60, 0.31), 0.184
Self-care	5–25	21.08 ± 4.669	24.20 ± 2.184	24.58 ± 1.281	< 0.001	47.669	< 0.001	Games–Howell	−3.503 (−4.72, −2.29), < 0.001	−0.380 (−1.13, 0.37), 0.448	−3.123 (−4.50, −1.74), < 0.001
Energy	3–15	8.85 ± 3.384	10.11 ± 3.570	10.39 ± 3.624	0.988	5.524	0.004	LSD	−1.537 (−2.45, −0.62), 0.001	−0.276 (−1.37, 0.81), 0.618	−1.261 (−2.47, −0.05), 0.041
Work productivity	3–15	9.95 ± 2.905	11.52 ± 2.626	12.40 ± 2.365	0.011	26.347	< 0.001	Games–Howell	−2.451 (−3.30, −1.60), < 0.001	−0.886 (−1.84, 0.07), 0.075	−1.564 (−2.69, −0.44), 0.004
Social roles	5–25	12.63 ± 5.383	15.56 ± 5.964	17.33 ± 5.793	0.719	19.597	< 0.001	LSD	−4.697 (−6.17, −3.22), < 0.001	−1.774 (−3.53, −0.02), 0.047	−2.923 (−4.87, −0.98), 0.003

SS-QOL, Stroke-Specific Quality of Life; MD, mean difference; CI, confidence interval.

Values are presented as mean ± standard deviation. Homogeneity of variances was assessed using Levene's test. LSD *post-hoc* comparisons were used for domains with homogeneous variances, whereas Games–Howell *post-hoc* comparisons were used for domains with unequal variances. No additional adjustment for multiple testing across domains was applied.

### 3.5 Multivariate logistic regression analysis of factors influencing latent classes

The three latent classes identified by LCA were treated as the dependent variable in the multinomial logistic regression model, with the Function Maintenance Group used as the reference category. Predictor coding and reference categories are shown in Table 1. Full model estimates, including non-significant associations, are presented in Table 1.

Compared with the Function Maintenance Group, participants aged 60–74 years (OR = 0.365, 95% CI: 0.185–0.718, *p* = 0.004), those with mild stroke severity (OR = 0.158, 95% CI: 0.064–0.393, *p* < 0.001), and those with sleep duration > 6 h (OR = 0.424, 95% CI: 0.231–0.779, *p* = 0.006) had lower odds of belonging to the Low Motor–Low Vitality Group. In contrast, participants with low social support (OR = 3.420, 95% CI: 1.158–10.103, *p* = 0.026) and moderate social support (OR = 1.994, 95% CI: 1.070–3.717, *p* = 0.030), compared with those with high social support, had higher odds of belonging to this group. Education level was not significantly associated with membership in the Low Motor–Low Vitality Group after adjustment.

For the Low Cognitive–Moderate Vitality, Psychological and Sensory Group, participants with primary school education or below had lower odds of belonging to this group than those with senior high school education or above (OR = 0.191, 95% CI: 0.043–0.859, *p* = 0.031). Age and sleep duration showed borderline associations with membership in this group, whereas stroke severity, social support, and junior high school education were not significantly associated with class membership after adjustment.

## 4 Discussion

### 4.1 Group heterogeneity in intrinsic capacity

This study identified three distinct intrinsic-capacity (IC) profiles among hospitalized older people with stroke, and most participants showed decline in at least one IC domain. These findings highlight substantial heterogeneity in multidomain IC impairment in this population.

Overall, 72.0% of participants experienced decline in at least one intrinsic-capacity domain, which is slightly higher than the 69.1% reported in general older populations (8). Older people with stroke often experience concurrent impairments in motor, cognitive, sensory, nutritional, and psychological function, which may contribute to reduced quality of life and functional decline (33). The three-class solution therefore provides a useful framework for characterizing these multidimensional patterns.

The Function Maintenance Group (55.1%) showed relatively preserved function across IC domains, although some decline remained, particularly in vitality. This finding suggests that even older people with stroke who appear functionally stable may still have early nutritional vulnerability requiring attention (34).

The Low Motor–Low Vitality Group (27.7%) was characterized by marked impairment in locomotion and vitality. These deficits may be mutually reinforcing, as mobility limitation can reduce daily activity and nutritional intake, while malnutrition may aggravate sarcopenia, weakness, and further mobility decline (34, 35).

The Low Cognitive–Moderate Vitality, Psychological, and Sensory Group (17.2%) showed prominent cognitive impairment together with moderate deficits in vitality, psychological status, and sensory function. This pattern is consistent with the high prevalence of post-stroke cognitive impairment and its frequent co-occurrence with nutritional problems, depressive symptoms, and sensory dysfunction (33, 36–40).

Taken together, these findings suggest that IC profiling may provide added value beyond isolated single-domain assessment by identifying co-occurring patterns of decline. In clinical practice, such profiling may help clinicians prioritize rehabilitation goals, tailor multidisciplinary care, and identify patients who require closer follow-up after hospitalization (50). For example, the Low Motor–Low Vitality Group may benefit most from integrated mobility and nutritional support, whereas the Low Cognitive–Moderate Vitality, Psychological, and Sensory Group may require greater emphasis on cognitive rehabilitation, psychological support, and sensory/environmental management. For the Function Maintenance Group, IC profiling may help support early prevention and continued monitoring before further decline emerges.

### 4.2 Influencing factors of intrinsic capacity latent classes

#### 4.2.1 Demographic characteristics

The multinomial logistic regression results showed that age, stroke severity, sleep duration, and social support were mainly associated with membership in the Low Motor–Low Vitality Group, whereas education level was associated with membership in the Low Cognitive–Moderate Vitality, Psychological, and Sensory Group. These findings suggest that different intrinsic-capacity profiles may have distinct correlates in hospitalized older people with stroke, which is broadly consistent with previous research (41).

Participants aged 60–74 years were less likely to be classified into the Low Motor–Low Vitality Group than those aged 75 years or older, suggesting that relatively younger stroke survivors tend to preserve better intrinsic capacity. Age-related decline in intrinsic capacity may reflect the combined effects of physiological aging, reduced physiological reserve, and accumulated disease burden (9, 49).

Education level was associated with membership in the Low Cognitive–Moderate Vitality, Psychological, and Sensory Group. In our data, participants with primary school education or below had lower odds of belonging to this group than those with senior high school education or above. This finding should be interpreted cautiously. Educational attainment may reflect not only health literacy and self-management capacity, but also cognitive reserve, which can modify how cognitive impairment manifests after stroke. Recent evidence from a pooled cohort study suggested that post-stroke cognitive trajectories vary by both education level and cognitive domain; compared with stroke survivors with lower educational attainment, those with higher education may have greater cognitive reserve, yet may also show steeper decline in specific domains such as executive function (42). Given the heterogeneity of post-stroke cognitive trajectories across educational strata and cognitive domains, future longitudinal studies using domain-specific cognitive measures are needed to clarify how education shapes intrinsic-capacity profiles after stroke.

Longer sleep duration (>6 h) was associated with lower odds of belonging to the Low Motor–Low Vitality Group and showed a borderline association with the Low Cognitive–Moderate Vitality, Psychological, and Sensory Group. Adequate sleep may support neuroplasticity, reduce fatigue, and promote recovery after stroke, whereas insufficient sleep may adversely affect physical, cognitive, and psychological function (43). These findings underscore the importance of identifying and managing sleep problems in older people with stroke during hospitalization and rehabilitation.

#### 4.2.2 Stroke severity and social support

Participants with mild stroke had substantially lower odds of belonging to the Low Motor–Low Vitality Group than those with moderate or severe stroke, indicating that worse neurological deficit is linked to poorer intrinsic-capacity status. This finding is consistent with prior studies (44). Neurological deficits affect core functions such as motor ability, cognition, swallowing, and sensory integration, which may in turn compromise daily functioning and quality of life and accelerate intrinsic-capacity decline (45). Routine evaluation of stroke severity and rehabilitation progress is therefore important for individualized care planning and targeted rehabilitation.

Lower levels of perceived social support were associated with higher odds of belonging to the Low Motor–Low Vitality Group. Compared with participants reporting high social support, those with low or moderate social support were more likely to fall into this more impaired profile. Social support may enhance coping ability, treatment adherence, emotional resilience, and engagement in rehabilitation, thereby helping preserve intrinsic capacity (46). These findings support the importance of strengthening family involvement, peer support, and communication between healthcare professionals and patients during stroke recovery (15, 46).

Notably, the factors associated with latent-class membership in this study included both relatively non-modifiable characteristics (such as age and educational attainment) and potentially modifiable factors (such as sleep duration and social support). This distinction is clinically meaningful because non-modifiable factors may help identify vulnerable subgroups, whereas modifiable factors may represent practical intervention targets during hospitalization and early recovery.

#### 4.2.3 Quality of life

Significant differences in stroke-specific quality of life were observed among the latent classes in 11 of the 12 SS-QOL domains, with no significant difference in personality. Overall, the Function Maintenance Group showed the most favorable quality-of-life profile, whereas the Low Motor–Low Vitality Group had the lowest scores across multiple domains. These findings further support the close relationship between intrinsic capacity status and quality of life in older people with stroke (47).

Pairwise comparisons showed that, compared with the Function Maintenance Group, the Low Motor–Low Vitality Group had significantly lower scores in energy, family roles, language, mobility, mood, self-care, social roles, cognition, upper extremity function, vision, and work productivity. This pattern suggests that combined decline in locomotion and vitality may have broad adverse effects on daily functioning, participation, and overall wellbeing, which is consistent with previous findings (48).

In contrast, compared with the Function Maintenance Group, the Low Cognitive–Moderate Vitality, Psychological, and Sensory Group showed significant disadvantages in cognition, family roles, and social roles. This pattern suggests that this profile may be associated not only with difficulties in cognitive functioning and family role performance, but also with reduced social participation in daily life. These findings are broadly consistent with previous evidence indicating that post-stroke multidomain deficits may substantially affect everyday functioning and quality of life (8).

### 4.3 Implications

Intrinsic-capacity (IC) profiling may have important practical and policy implications for the care of people with stroke. In clinical practice, IC profiling may help clinicians rapidly identify heterogeneous care needs and prioritize domain-focused rehabilitation and nursing strategies across subgroups of people with stroke. It may also support more structured multidisciplinary communication by helping physicians, nurses, therapists, dietitians, and family caregivers focus on the domains most likely to limit recovery and quality of life. At the policy level, incorporating routine IC screening and profiling into inpatient stroke care and discharge planning may support risk stratification and facilitate targeted referral pathways, including rehabilitation, nutrition, sleep management, and social-support services. In the longer term, such an approach may contribute to more efficient allocation of rehabilitation resources and more individualized continuity-of-care models for older people with stroke.

### 4.4 Limitations

This study has several limitations. First, the cross-sectional design precludes any inference of causality regarding the associations among intrinsic-capacity profiles, influencing factors, and stroke-specific quality of life. Longitudinal studies are needed to examine how intrinsic-capacity profiles evolve over time and how they relate to subsequent recovery and quality-of-life outcomes.

Second, this was a single-center study conducted in a tertiary hospital, which may limit the generalizability of the findings to other settings, such as community-based populations, primary care institutions, or hospitals in other regions. In addition, although participants were identified as being beyond the acute stage on the basis of medical-record documentation and clinical stability, exact time since stroke onset was not consistently available for all participants. Therefore, stroke stage was determined pragmatically rather than by a standardized onset-to-assessment time threshold, which may have introduced some imprecision in stage classification.

Third, after latent class analysis, each participant was assigned to the class with the highest posterior probability for subsequent analyses. Although the entropy and posterior probabilities indicated good classification quality, classification uncertainty was not directly incorporated into the regression and ANOVA analyses, which may have led to some degree of misclassification bias.

Fourth, several measures relied on questionnaire-based or interviewer-administered assessments and may therefore have been subject to reporting bias or information bias. In particular, some psychosocial and lifestyle variables, such as perceived social support and sleep duration, may be influenced by subjective perception.

Finally, although we adjusted for several demographic, clinical, and psychosocial variables, residual confounding cannot be ruled out. Additional multicenter longitudinal studies with more detailed stroke-related information and domain-specific cognitive assessment are warranted to validate and extend these findings.

## 5 Conclusion

This study identified three distinct intrinsic-capacity profiles among hospitalized older people with stroke, and these profiles were associated with different levels of stroke-specific quality of life. Age, educational level, sleep duration, stroke severity, and social support were important factors associated with profile membership. These findings suggest that intrinsic-capacity profiling may help identify heterogeneous functional needs in this population and support more individualized rehabilitation and nursing strategies. In clinical practice, targeted, domain-focused interventions may be particularly important for people with stroke who are older, have lower educational attainment, shorter sleep duration, more severe neurological deficits, or lower social support. From a broader practice and policy perspective, integrating routine IC screening and profiling into inpatient stroke care and discharge planning may improve risk stratification, continuity of care, and allocation of supportive rehabilitation resources for high-risk older people with stroke.

## Data Availability

The raw data supporting the conclusions of this article will be made available by the authors, without undue reservation.
